# Two-Stage Polyhydroxyalkanoates (PHA) Production from Cheese Whey Using *Acetobacter pasteurianus* C1 and *Bacillus* sp. CYR1

**DOI:** 10.3390/bioengineering8110157

**Published:** 2021-10-24

**Authors:** Young-Cheol Chang, Motakatla Venkateswar Reddy, Kazuma Imura, Rui Onodera, Natsumi Kamada, Yuki Sano

**Affiliations:** 1 Course of Chemical and Biological Engineering, Division of Sustainable and Environmental Engineering, Muroran Institute of Technology, Hokkaido 050-8585, Japan; 21041006@mmm.muroran-it.ac.jp (K.I.); rui.onodera08@gmail.com (R.O.); 21041034@mmm.muroran-it.ac.jp (Y.S.); 2 Course of Biosystem, Department of Applied Sciences, Muroran Institute of Technology, Hokkaido 050-8585, Japan; 18023034@mmm.muroran-it.ac.jp; 3 Center for Biotechnology and Interdisciplinary Studies, Rensselaer Polytechnic Institute, Troy, NY 12180, USA; mvr_234@yahoo.co.in or

**Keywords:** cheese whey, acetic acid, *Acetobacter pasteurianus* C1, *Bacillus* sp. CYR-1, PHA, PHB

## Abstract

Cheese whey (CW) can be an excellent carbon source for polyhydroxyalkanoates (PHA)-producing bacteria. Most studies have used CW, which contains high amounts of lactose, however, there are no reports using raw CW, which has a relatively low amount of lactose. Therefore, in the present study, PHA production was evaluated in a two-stage process using the CW that contains low amounts of lactose. In first stage, the carbon source existing in CW was converted into acetic acid using the bacteria, *Acetobacter pasteurianus* C1, which was isolated from food waste. In the second stage, acetic acid produced in the first stage was converted into PHA using the bacteria, *Bacillus* sp. CYR-1. Under the condition of without the pretreatment of CW, acetic acid produced from CW was diluted at different folds and used for the production of PHA. Strain CYR-1 incubated with 10-fold diluted CW containing 5.7 g/L of acetic acid showed the higher PHA production (240.6 mg/L), whereas strain CYR-1 incubated with four-fold diluted CW containing 12.3 g/L of acetic acid showed 126 mg/L of PHA. After removing the excess protein present in CW, PHA production was further enhanced by 3.26 times (411 mg/L) at a four-fold dilution containing 11.3 g/L of acetic acid. Based on Fourier transform infrared spectroscopy (FT-IR), and ^1^H and ^13^C nuclear magnetic resonance (NMR) analyses, it was confirmed that the PHA produced from the two-stage process is poly-β-hydroxybutyrate (PHB). All bands appearing in the FT-IR spectrum and the chemical shifts of NMR nearly matched with those of standard PHB. Based on these studies, we concluded that a two-stage process using *Acetobacter pasteurianus* C1 and *Bacillus* sp. CYR-1 would be applicable for the production of PHB using CW containing a low amount of lactose.

## 1. Introduction

The replacement of traditional plastics by bioplastics is gaining interest in the increasing of the sustainability of the polymer industry. One of the most promising groups of bioplastics are polyhydroxyalkanoates (PHA) [1]. However, the popularization of PHA has been limited by their production cost, which remains rather high, with raw materials responsible for most of the cost [2]. Therefore, in order to make PHA production more feasible for industrial application, different inexpensive substrates, such as molasses, sugars, oils, fatty acids, glycerol, organic matter from waste, starch-based materials, cellulosic materials, and hemi-cellulosic materials have been tested [3]. Reports are also available for the valorization of cheese whey (CW) into PHA [4]. CW is the liquid part of milk that is separated from the curd at the beginning of cheese manufacture, is available in large amounts as a by-product, and contains fermentable nutrients, such as lactose, lipids, and soluble proteins [4]. CW separated in cheese production corresponds to about 90% in raw milk; therefore, its disposal as waste causes serious pollution problems in the surrounding environment [5,6]. In Japan, the number of cheese-producing factories is steadily increasing. Compared to 2006, the number of factories was tripled (319 factories, excluding major dairy companies) in 2018 (Japan Agriculture & Livestock Industries Corporation). However, the majority of CW is discarded into the environment, owing to expensive treatment processes. Therefore, the conversion of CW into a useful product, PHA, is a good option to reduce the environmental burden.

Reports are available for PHA production using CW and various microorganisms, such as *Methylobacterium* sp. ZP24, *Bacillus megaterium* CCM2037, *Azohydromonas lata* DSM1123, *Hydrogenophaga pseudoflava* DSM1034, and *Haloferax mediterranei* DSM1411 [5]. Most of the studies used CW, which contains high amounts of lactose. Lactose present in CW converts into glucose-galactose by the action of β-galactosidase enzymes, and then converts further into PHA [5]. For achieving an economically competitive production of PHA from CW, there is a need to discover the wild-type bacterial species that can directly produce high levels of PHA. However, currently, only a few wild-type bacteria are available that can directly produce PHA from whey lactose [7]. Recombinant *E. coli* is widely used for PHA production, because many wild-type bacteria cannot directly metabolize the CW, due to the absence of β-galactosidase enzymes [8].

While the genetic engineering method is a versatile tool for PHA production, it requires more controlled production plants, which increases the cost [9]. Amaro et al. suggested that using CW and wild-type strains will reduce the production cost [9]. Hence, the present studies were carried out using CW and wild-type bacterial strains for the production of PHA. 

PHA has been extensively studied as an alternative to petroleum-based plastics [10]. However, it has not yet been in practical use because of its high production cost, primarily owing to the high cost of substrates (11% of the total production cost) that are used for the growth of microorganisms [10]. For this reason, in our previous study, we examined the capability of strain CYR1 in producing PHA using raw CW as a feed stock. However, the production yield was not as high as we expected, at 0.15 g/L PHB of the dry cell weight (DCW) [10]. On the other hand, various fatty acids (short and medium chain fatty acids) and their subsequent conversion into PHA were evaluated in order to investigate the possibility of using wastewater containing fatty acids. As a result, acetic acid was the most efficient carbon source for PHA production among the tested fatty acids [11]. Obviously, CW is still a very attractive feedstock for the production of PHA. Thus, to improve the capability of PHA production, we designed a two-stage process: CW was converted to acetic acid using *Acetobacter pasteurianus* C1 in the first stage, and, in the second stage, acetic acid produced from CW was converted to PHA using *Bacillus* sp. CYR-1. Firstly, acetic-acid-producing bacteria (*Acetobacter pasteurianus* C1) was isolated, and the culture conditions for acetic acid production from CW were determined. In the previous study, we did not know whether strain CYR1 utilizes the lactose that is in CW. Therefore, a utilization test of lactose using strain CYR1 and *Acetobacter pasteurianus* C1 was performed. Further experiments were conducted for PHA production with acetic acid produced from CW. Furthermore, the PHA production was also evaluated with pretreated CW (after removing excess protein). The consumption of acetic acid and the concentration of the PHA produced were analyzed with high-performance liquid chromatography (HPLC). Finally, the functional groups, primary structure, higher-order structure, and thermal properties of the produced poly-β-hydroxybutyrate (PHB) from the two-stage process were analyzed using Fourier transform infrared spectroscopy (FT-IR), ^1^H ^13^C nuclear magnetic resonance (NMR), and thermogravimetric analysis (TGA), respectively.

## 2. Materials and Methods

### 2.1. Biocatalyst

Bacteria *Acetobacter pasteurianus* C1 isolated in the present study and *Bacillus* sp. CYR1 were used to produce acetic acid and PHA, respectively. Our previous studies have proven that CYR1 strain can utilize diverse types of fatty acids, such as C2–C6, for its growth [10]. 

### 2.2. Isolation of Acetic-Acid-Producing Bacteria and Optimum Conditions for Acetic Acid Production 

Fermented food waste was used for the isolation of acetic-acid-producing bacteria. The liquid (leachate) obtained from the food waste was diluted at 10 folds with saline solution (NaCl, 0.9%), spread on agar plates, and incubated under aerobic conditions at 30 °C for several days. Obtained bacterial colonies were separated based on the color, shape, and size, and each individual colony was grown on new agar plates until a pure culture was obtained. Morphological characterization of pure bacterial colonies was carried out by staining with a Gram stain kit (Becton Dickinson Company, Tokyo, Japan ). A total of 40 pure bacterial colonies were obtained, and the purity of each isolate was determined using an optical microscope (DA1-180M, AS ONE, Osaka, Japan). 

Isolated bacteria (40 pure colonies) were separately pre-grown for 72 h on mineral salt medium (MS medium) containing yeast extract (10 g/L) as a sole nutrient source. The medium pH was adjusted to 5.8 and sterilized in an autoclave for 15 min at 121 °C. Pre-cultures were harvested by centrifugation (8000× *g*, 4 °C, 10 min), and obtained pellet was washed two times to remove the remaining carbon sources and suspended in MS medium, which contains 36 g/L of glucose, 10 g/L of yeast extract, and ethanol (7.76 g/L) in serum bottles capped with autoclave-sterilized air-permeable silicone plugs, which were then incubated aerobically with orbital shaking at 120 rpm for 5 days at 30 °C for acetic acid production. The optimum conditions, i.e., initial cell concentration, pH (3 to 8), temperature (25 to 42°C), ethanol concentration (0 to 77.63 g/L), and rotation speed, were also determined to produce acetic acid using the MS medium. Experiments were conducted for 4 days under shaking at 100 rpm and pH 7.0 in 50 mL serum bottles. Ethanol concentration experiments were conducted for 10 days. Agar plates were prepared by dissolving polypeptone (5 g), yeast extract (5 g), glucose (5 g), MgSO_4_·7H_2_O (1 g), and agar (20 g) in one liter of distilled water. One liter of MS medium contained the following composition: 1.0 g K_2_HPO_4_, 0.05 g NaCl, 0.2 g MgSO_4_·7H_2_O, 0.05 g CaCl_2_, 0.0083 g FeCl_3_·6H_2_O, 0.014 g MnCl_2_·4H_2_O, 0.017 g NaMoO_4_·2H_2_O, and 0.001 g ZnCl_2_.

Among 40 pure colonies, the highest acetic-acid-producing bacteria were identified by 16S rDNA analysis. The isolate was grown on a yeast extract medium, and total genomic DNA was extracted using the achromopeptidase (Fujifilm Wako Pure Chemical, Japan) in accordance with the protocol provided by the manufacturer. Extracted bacterial genomic DNA was amplified by polymerase chain reaction (PCR) with a pair of universal primers: 10f (5′-GAGTTTGATYCTGGCTCAG-3′) and 1510r (5′-GGTTACCTTGTTACGACTT-3′), under standard conditions. PCR was performed for 2 min at 94 °C, followed by 30 amplification cycles of 45 s at 94 °C, 30 s at 52 °C, and 90 s at 72 °C, with a final cycle for 20 min at 72 °C. Amplified PCR products were purified with a QIAquick PCR purification kit (Qiagen, Hilden, Germany). Sequences were determined using an ABI prism 3130 Sequencer (Applied Biosystems, CA, USA), and the sequence products were assembled with ChromasPro 2.1 (Technelysium, Tewantin, Australia). Obtained DNA sequences were compared using Basic Local Alignment Search Tool (BLAST) search program with the National Center for Biotechnology Information (NCBI) gene bank database (www.ncbi.nlm.nih.gov, accessed on 28 September 2021). DNA sequencing was performed according to the previously reported method [12,13]. 

### 2.3. Acetic Acid Production with CW

CW was collected from a cheese-producing factory in Hokkaido, Japan, and stored in a darkroom at 4 °C. Even though it had high chemical oxygen demand (COD, 107,000 mg/L) and total nitrogen (TN, 1380 mg/L), it contained low lactose (approximately 4.0 g/L) concentration. The addition of ethanol is indispensable for the current acetic acid fermentation [14]. The CW was diluted 2-fold with deionized water, and pH was adjusted to 5.5 or 6.6 with 1 N HCl or 1 N NaOH, which was added (200 mL) into 500 mL Erlenmeyer flask, and autoclaved (121 °C, 15 min). After cooling, 2 mL of pre-cultivated cells and 2 mL of 99.5% ethanol were added, and the flasks were capped with autoclave-sterilized air-permeable silicone plugs and maintained under shaking (100 rpm) at 30 °C in an incubator. Control experiments were carried out without adding C1 strain. Samples were collected at different time intervals aseptically on a clean bench to measure the pH, acetic acid concentration, and ethanol concentration. Experiments were stopped when ethanol concentration reached below 0.3 g/L, because acetic acid production is dependent on ethanol consumption, as confirmed in our preliminary experiments.

### 2.4. Lactose Utilization Experiments 

PHA-producing bacteria (*Bacillus* sp. CYR1) and acetic-acid-producing bacteria (C1 strain) were examined regarding whether these bacteria can utilize lactose or not. Acetic acid, glucose, yeast extract, lactose, and poly peptone, each at 5 g/L concentration, as well as 0.2 mL of ethanol, were added in MS medium. Medium pH was adjusted to 7.0, and 20 mL of medium was dispensed into a 50 mL serum bottle and autoclaved. Ethanol was added after autoclave. Bacteria were added in each serum bottle except blank, and the serum bottles were incubated aerobically at 30 °C with orbital shaking at 120 rpm for 7 days and 11 days for CYR1 and C1 strains, respectively. All of the conditions were tested as biological triplicates (three serum bottles, *n* = 3) within a single experiment.

### 2.5. PHA Production without Pre-Treatment of CW and Quantification

Two-stage PHA production experiments were performed with *Acetobacter pasteurianus* C1 and *Bacillus* sp. CYR-1 using acetic acid obtained from CW. The initial concentration of acetic acid and total nitrogen from the CW fermentation step was 24.6 g/L and 0.853 g/L, respectively. To lower the concentration of total nitrogen, dilutions were made (4-fold, 10-fold, and 20-fold) and used for the PHA production. The two-stage process was carried out in different Erlenmeyer flasks. Experiments were also carried out using the CW without dilution. PHA production experiments were conducted in 1 L Erlenmeyer flasks that contain 500 mL of diluted CW containing acetic acid after adjusting the pH to 7.0. Pre-grown CYR1 cultures were added to each flask and incubated for 60 h under shaking (100 rpm) at 30 °C. For diluted CW, the growth curve was measured using a spectrophotometer (UV-1800, Shimadzu, Kyoto, Japan ) at OD_600_. Each diluted CW without addition of bacteria (1 mL) was used as a blank [10]. We did not adopt the dry weight method because the variation of each sample was significant due to the amount of whey being much higher than that of the cells.

PHA separation from culture was performed as mentioned by Law and Slepecky [15], as well as by the Soxhlet extraction method. Centrifugation (8000× *g* for 10 min at 4 °C) was used to separate the cells, and cell disruption was carried out using a Branson ultrasonic disrupter (Sonifer 250, Danbury, CT, USA) at three-minute flash for 30 min at output—3 and duty cycle—50%. Cell debris was removed by centrifugation at 3000× *g* for 20 min. Sodium hypochlorite (4%) was used for suspension of biomass pellet, and the solution was incubated for 4 h at room temperature. The resulting solution (the pellet with lysed cells) was centrifuged, and the supernatant was discarded. The lysed cells were washed with acetone and ethanol, and they were liquefied in hot CHCl_3_ and filtered. The filtrate was concentrated using a rotary evaporator (EYELA N-1000, Tokyo Rikakikai, Japan), followed by drying until obtaining a persistent weight. This procedure was adopted for the evaluation of dry cell mass. To enhance the PHA extraction effect, the Soxhlet extraction method was adopted. After centrifugation, the biomass pellet was disrupted with a Branson ultrasonic disrupter. Cell debris was removed by centrifugation at 8000× *g* for 10 min, and the pellet was placed in an extraction thimble. CHCl_3_ was added to the round-bottom flask, and Soxhlet extraction was carried out for 24 h. 

### 2.6. Effect of Pre-Treatment of CW for the Production of PHA

After finishing the above-mentioned two-stage PHA production experiments, we realized that many studies removed excess protein present in CW for the PHA production experiments [9,16,17]. To remove excess protein present, CW was acidified at pH 4.0 with 1.0 M H_2_SO_4_ [16]. The solution was autoclaved at 121 °C for 15 min and centrifuged at 8000× *g* in sterilized tubes for 10 min to remove aggregates [17]. The supernatant (whey supernatant) was passed through ADVANTEC No. 2 filters (ADVANTEC, City, Japan). PHA production was performed in a two-stage process with *Acetobacter pasteurianus* C1 and *Bacillus* sp. CYR1.

### 2.7. PHB Identifivation

The FT-IR spectrum of PHB produced by CYR-1 was measured by attenuated total reflection using a Nicolet 6700 FT-IR spectrometer (Thermo Fisher Scientific, Waltham, MA, USA). ^1^H (300 MHz) and ^13^C (75 MHz) NMR spectra were recorded on an Oxford YH300 NMR spectrometer (Oxford instrument, Oxford, UK) at 20 °C. The NMR sample was dissolved in deuterated chloroform (CDCl_3_, Sigma-Aldrich), and impurities were removed by filtration with cotton. TGA (TGA 2050, TA Instruments, New Castle, DE, USA) was used to determine the decomposition temperature (Td) of PHB.

### 2.8. Analysis

COD_Mn_ was measured using the permanganate method with a COD-60A meter (DKK-TOA Corp., Shinjuku-ku, Japan). Total nitrogen (TN) was measured using a DRB 200 dual block (Hach, Loveland, CO, USA) and DR 900 multiparameter portable colorimeter (Hach, Loveland, CO, USA). Spectrometric analysis was conducted to determine the PHB concentration. The extracted PHB by using the Soxhlet method was dissolved in 1 mL of sulfuric acid (36 N), and the resultant solution was heated at 100 °C in a dry thermos bath for 60 min. The addition of sulfuric acid converted PHB into crotonic acid. The solution was cooled and diluted 50 times using sulfuric acid (0.01 N). The solution was filtrated and analyzed by HPLC (Shimadzu, Kyoto, Japan) with an SPD-10AV UV/Vis detector and a Shim-pack SCR-102 (H) column (Shimadzu, Kyoto, Japan). Filtered and degassed 5 mmol/L perchloric acid was used as mobile phase at a flow rate of 1.5 mL/min. The column was maintained at a temperature of 40 °C in a thermostat chamber. The absorbance was measured at 210 nm for determining the PHB concentration. A standard curve was prepared using pure PHB (Sigma-Aldrich). The concentrations of acetic acid and lactose at different time intervals were analyzed on HPLC (Shimadzu) with an RI detector and Shim-pack SCR-102 (H) column (Shimadzu, Kyoto, Japan). Samples collected for HPLC analysis were acidified with phosphoric acid (10%, w/v) to stop the biological reaction and centrifuged at 8000× *g* for 10 min. The resulting supernatant was filtered and analyzed directly by HPLC. Filtered and degassed 5 mmol/L perchloric acid was used as mobile phase at a flow rate of 1.5 mL/min. The column was maintained at a temperature of 40 °C in a thermostat chamber. 

## 3. Results and Discussion

### 3.1. Acetobacter Pasteurianus C1 Strain 

**Figure 1:**
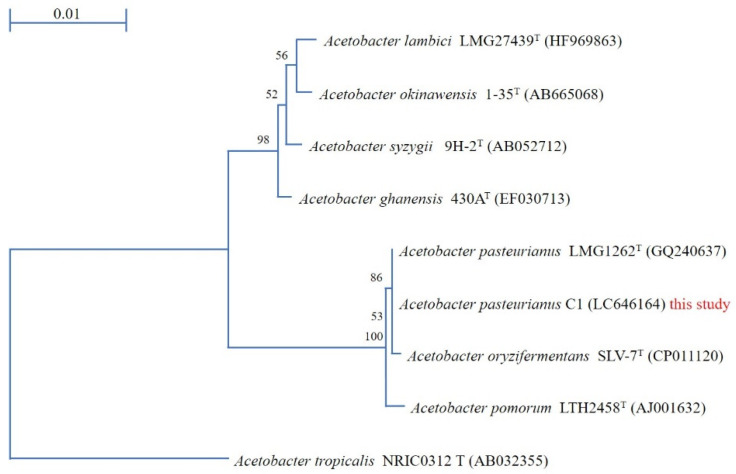
A neighbor-joining tree showing the phylogenetic relationship of the 16S rDNA sequence of C1 strain with related organisms. Bootstrap values of 100 analyses are shown at the branch point.

Acetic acid production studies were conducted using the 40 isolated bacterial strains; among them, three colonies produced good amounts of acetic acid, but colony #36 produced the highest amount of acetic acid. Hence, genomic DNA was isolated from this strain and 16S rDNA sequence analysis was carried out. The gene sequence analysis showed that the strain has a 100% identity with *Acetobacter pasteurianus* LMG 1262^T^ (Figure 1), so strain #36 was named as *Acetobacter pasteurianus* C1, and its 16S rDNA sequence was deposited in the DNA Data Bank of Japan (DDBJ, Accession number LC646164). Below is the phylogenetic tree constructed based on the BLAST homology search for the International Nucleotide Sequence Databases.

It is a Gram-negative, rod-shaped bacteria, commonly associated with plants and plant products, and is widely used in the production of fermented foods, such as kefir and vinegar [18,19]. Acetic acid production using wild and engineered *Acetobacter pasteurianus* strains (*A. pasteurianus* SKU1108, *A. pasteurianus* TI and TH-3 (thermo-adapted strain SKU1108), *A. pasteurianus* CICIM B7003-2, *A. pasteurianus* CWBI-B419, *A. aceti* subs. *Xylinum* NBI1002, *A. aceti* subs. *aceti* 1023, *A. aceti* M23) and *Gluconacetobacter* bacterial strains (*G. europaeus* DES11-DSM 6160, *G. europaeus* V3 and JK2, *G. intermedius* JK3, *G. entanii* LTH 4560T) have been well-documented by Gullo et al. [20].

### 3.2. Optimum Conditions for the Production of Acetic Acid

**Figure 2:**
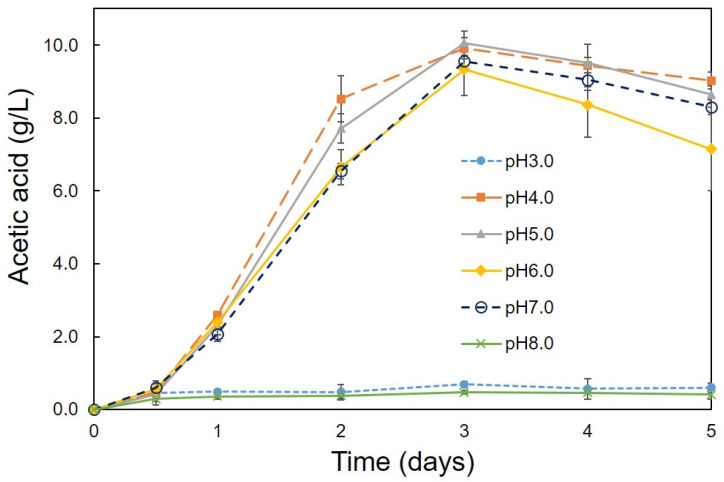
Evaluation of pH values for acetic acid production. Cultivation was conducted at 30 °C with initial cell concentration of 0.45 g/L, ethanol concentration of 7.76 g/L, and at the rotation speed of 100 rpm.

**Figure 3:**
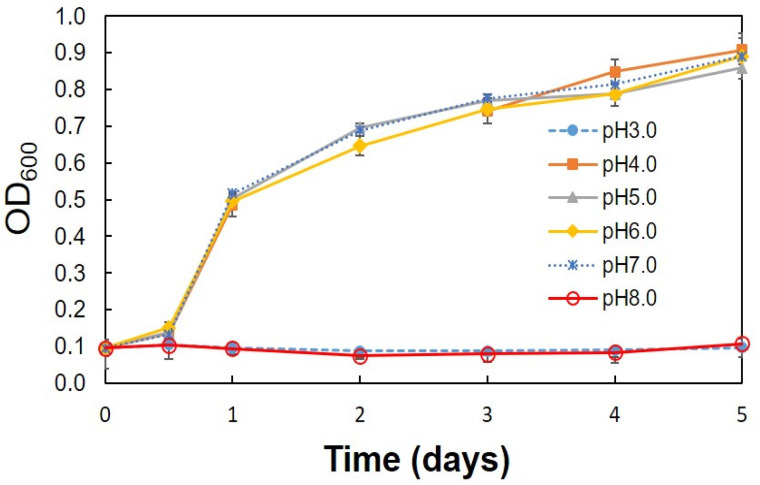
Growth curve of strain C1 at different initial pH values. Cultivation was conducted at 30 °C with initial cell concentration of 0.45 g/L, ethanol concentration of 7.76 g/L, and at the rotation speed of 100 rpm.

**Figure 4:**
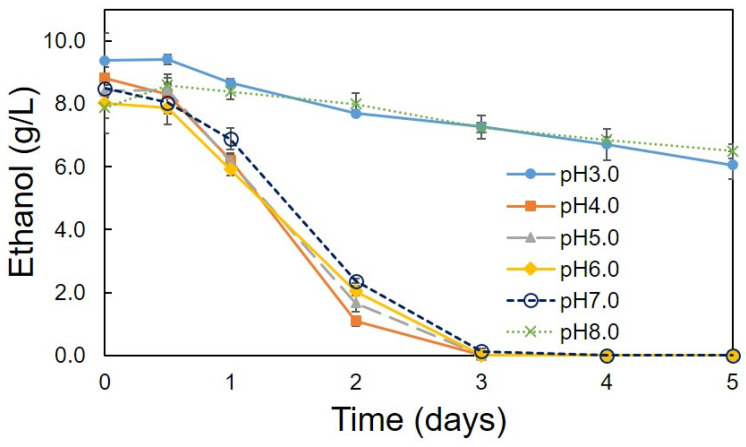
Evaluation of pH values for ethanol utilization. Cultivation was conducted at 30 °C with initial cell concentration of 0.45 g/L and at the rotation speed of 100 rpm. Ethanol (7.76 g/L) was added to the cultures at different pH values.

**Figure 5:**
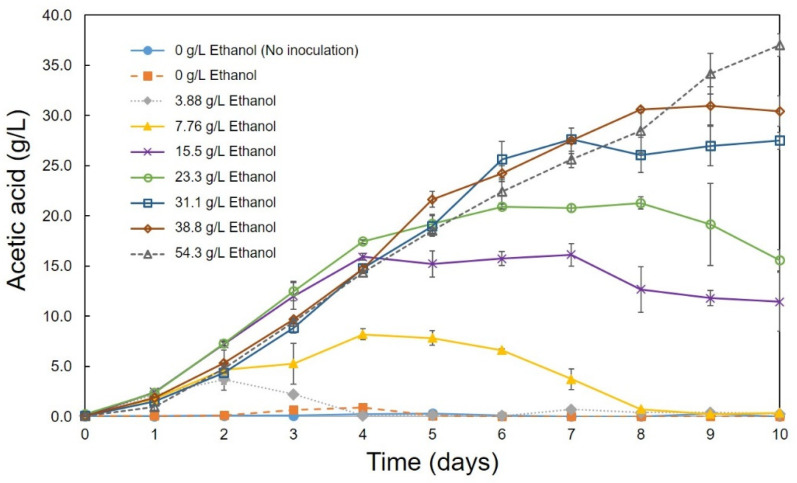
Acetic acid production at different initial ethanol concentrations. Cultivation was conducted at 30 °C with initial cell concentration of 0.45 g/L and at the rotation speed of 100 rpm.

**Figure 6:**
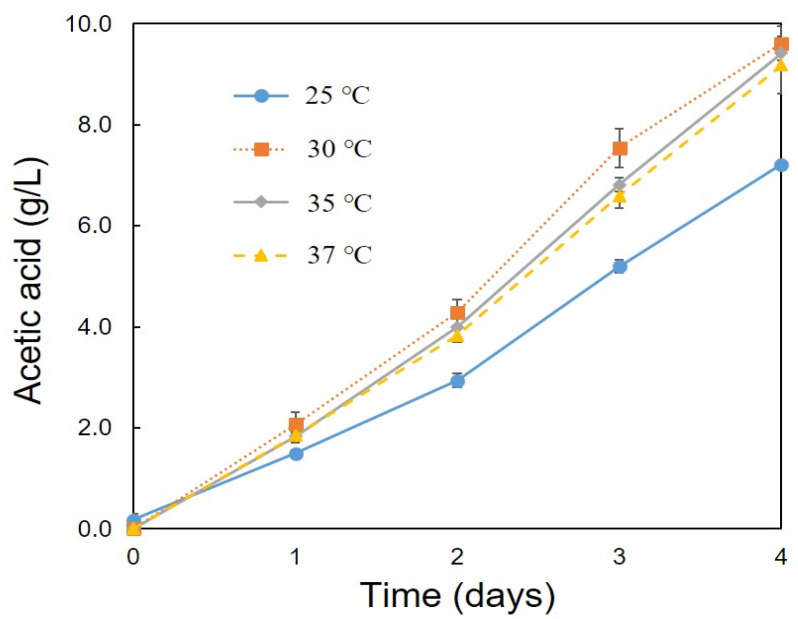
Optimal temperature conditions for acetic acid production. Cultivation was conducted with initial cell concentration of 0.45 g/L, ethanol concentration of 7.76 g/L, and at the rotation speed of 100 rpm.

**Figure 7:**
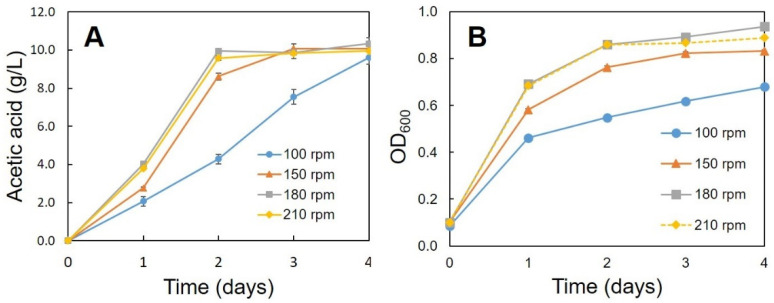
Acetic acid production (**A**) and growth curve of strain C1 (**B**) at different rotation speeds. Cultivation was conducted at 30 °C with initial cell concentration of 0.45 g/L and ethanol concentration of 7.76 g/L.

Optimum conditions (pH, temperature, ethanol concentration, rotation speed) for the production of acetic acid were determined using the C1 strain. A high amount of acetic acid production (10.05 g/L) was observed at pH 4.0 and 5.0 (Figure 2). Strain C1 produced acetic acid between pH 4.0 and 7.0, but it was unable to produce at pH 3.0 and 8.0 (Figure 2). At pH 3 and 8 lower growth was observed, (Figure 3), but, slight decrease in ethanol concentration at pH 3 and 8 (Figure 4) was observed it might be due to the evaporation of ethanol. A relatively similar cell growth of strain C1 was observed between pH 4 and pH 7. A rapid initial cell growth was observed at pH 4, 5 and 7 (Figure 3). Decrements in the ethanol concentration under different pH conditions were determined (Figure 4). Ethanol was completely utilized within 3 days at pH 4, 5, 6, and 7. The effect of the optimum ethanol concentration on acetic acid production was also examined. Acetic acid production was not observed without the addition of ethanol, and also at higher (77.63 g/L) ethanol concentrations (data not shown), whereas a higher acetic acid production (37.02 g/L) was observed at 54.34 g/L of ethanol (Figure 5). Regarding temperature, the C1 strain showed a higher acetic acid production (9.61 g/L) at 30 °C (Figure 6), and no production at above 40 °C (data not shown). The acetic acid production (Figure 7A) and growth curve of strain C1 (Figure 7B) at different rotation speeds were determined. The highest acetic acid production and cell growth were obtained at the rotation speed of 180 rpm. After the optimization of all of the conditions, the C1 strain showed a higher acetic acid production (37.02 g/L), with an initial cell concentration of 0.45 g/L, ethanol concentration of 54.34 g/L, and at the rotation speed of 180 rpm (data not shown). The acetic-acid-producing ability of the C1 strain was comparable to other wild-type strains (Table 1). 

Ndoye et al. reported that *A. pasteurianus* CWBI-B419 produced 16 g/L of acetic acid under the optimum conditions (38 °C, ethanol concentration of 19.4 g/L, and the rotation speed of 130 rpm) [21]. Kanchanarach et al. reported that *A. pasteurianus* MSU10 and SKU1108 produced 25 g/L and 35 g/L of acetic acid at 37 °C, with an ethanol concentration of 38.82~46.58 g/L and a rotation speed of 200 rpm [22]. Perumpuli et al. reported that *A. pasteurianus* SL13E-2, SL13E-3, and SL13E-4 produced more than 40 g/L of acetic acid at 37 °C, an ethanol concentration of 46.58 g/L, and at 200 rpm [23]. Es-sbata et al. reported that *A. malorum* strains produced more than 40 g/L of acetic acid at 37 °C [24]. Kadere et al. isolated several Acetobacter and Gluconobacter strains. The isolated Acetobacter and Gluconobacter strains both showed growth at 25, 30, and 40 °C and at pH 7.0 and 4.5, whereas there was no growth at 45 °C, and pH 2.5 and 8.5 [25]. Iino et al. reported that *G. kakiaceti* sp. I5-1^T^ and G5-1 showed growth at pH 3.5–8.0, whereas there was no growth at pH 3.0 and 8.5 [26]. Saeki et al. reported that *A.* *rancens* SKU 1102 produced approximately 43.0 g/L of acetic acid at 38 °C, with an ethanol concentration of 31.05 g/L and at 220 rpm, and with a supplement of acetic acid (10 g/L) [27]. Vashisht et al. reported that *A. pasteurianus* SKYAA25 produced 52.4 g of acetic acid using 100 g of dry matter of apple pomace at pH 5.5, temp 37 °C, ethanol concentration of 62.10 g/L, and at 180 rpm [28]. Chen et al. reported that *A. pasteurianus* AAB4 produced 42 g/L of acetic acid at 37 °C, an ethanol concentration of 77.63 g/L, and at 180 rpm [29]. Wu et al. reported that *A.* *pasteurianus* CICC 20001 produced 48.24 g/L of acetic acid in a 15 L stir tank reactor at 32 °C, an ethanol concentration of 35.70 g/L, and at 180 rpm, and with a supplement of acetic acid (12 g/L) [30]. Engineered strains *A. aceti* subsp. *Xylinum* NBI2099 (pAL25) and *A. pasteurianus* CICIM B7003-2 produced higher amounts of acetic acid, at 96.6 g/L and 90 g/L of acetic acid, respectively, than wild-type strains [31,32]. In order to compare the acetic acid production ability with other Acetobacter, *Acetobacter pasteurianus* (NBRC105185) was purchased from National Institute of Technology and Evaluation, Japan, and experiments were conducted for 10 days under similar conditions using both the strains in separate flasks. *Acetobacter pasteurianus* produced 33.9 g/L, whereas our strain C1 produced 37.02 g/L of acetic acid. Therefore, it can be concluded that the acetic acid production ability of the C1 strain is greater than that of *Acetobacter pasteurianus* (NBRC105185).

**Table 1:** Overview of optimum conditions reporting acetic acid production using pure microbial cultures.

Strain	Acetic Acid (g/L)	pH	Temperature (°C)	Ethanol (g/L)	Rotation Speed (rpm)	References
*Acetobacter pasteurianus* CWBI-B419	16	n.a	38	19.4	130	[21]
*Acetobacter pasteurianus* MSU10	25	n.a	37	38.82–46.58	200	[22]
*Acetobacter pasteurianus* SKU1108	35	n.a	37	38.82–46.58	200	[22]
*Acetobacter pasteurianus* SL13E-2	40	n.a	37	46.58	200	[23]
*Acetobacter pasteurianus* SL13E-3	40	n.a	37	46.58	200	[23]
*Acetobacter pasteurianus* SL13E-4	40	n.a	37	46.58	200	[23]
*Acetobacter malorum*	40	n.a	37	n.a	n.a	[24]
*Acetobacter rancens* SKU 1102	43.0	n.a	38	31.05	220	[27]
*Acetobacter pasteurianus* SKYAA25	52.5 g/100 g of dry substrate	5.5	37	62.10	180	[28]
*Acetobacter pasteurianus* AAB4	42	n.a	37	77.63	180	[29]
*Acetobacter pasteurianus* CICC 20001	48.24	n.a	32	35.70	180	[30]
*Acetobacter pasteurianus* C1	37.02	4–5	30	54.34	180	This study

n.a: not available.

All of the conditions were tested as biological triplicates (three serum bottles, *n* = 3) within a single experiment. The data were presented as mean ± standard deviation.

### 3.3. Lactose Utilization 

**Figure 8:**
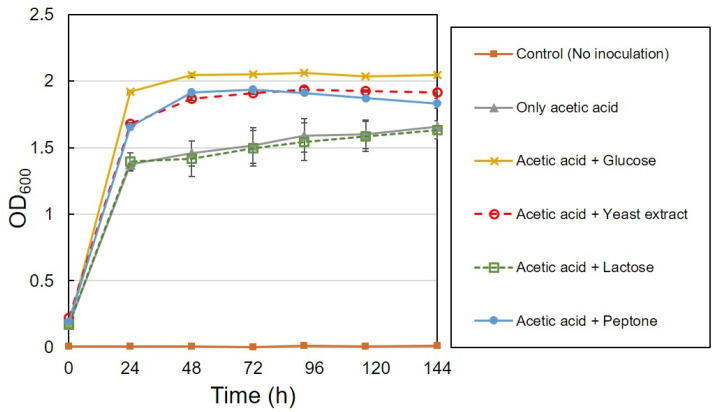
Bacterial growth curve using strain CYR1 with different carbon and nutrient sources. Cultivation was conducted at 30 °C, pH 7.0, ethanol concentration of 7.76 g/L, and at the rotation speed of 120 rpm.

**Figure 9:**
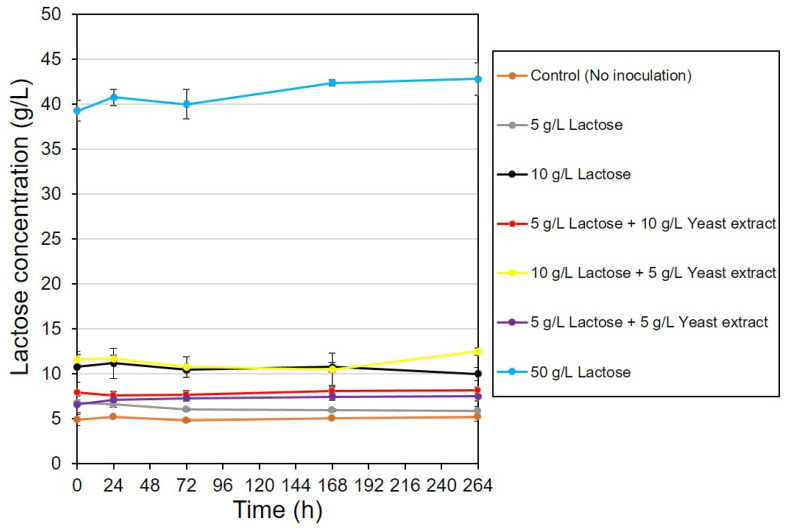
Lactose utilization experiments by strain C1 using various carbon sources. Cultivation was conducted at 30 °C, pH 7.0, ethanol concentration of 7.76 g/L, and at the rotation speed of 120 rpm.

Various carbon sources (acetic acid, glucose, lactose) and nutrient sources (peptone, yeast extract) at different combinations were used for the growth of CYR1, and the growth was measured with a spectrophotometer by taking the culture absorbance at OD_600_, as shown in Figure 8. OD_600_ increased with all of the conditions except the control. Control experiments were conducted without adding the CYR1 strain. Bacteria grown with the medium, which contained glucose and acetic acid as carbon sources, showed the highest growth (OD_600_ 2.05). There was no difference in the growth of bacteria when they were grown with a medium that contained only acetic acid, or with the combination of acetic acid and lactose. Lactose utilization was examined also using the *Acetobacter pasteurianus* C1 strain. However, as shown in Figure 9, a decrement in the lactose concentration was not observed in all experiments regardless of the presence or absence of a co-substrate. Thus, it can be understood that the CYR1 and C1 strains are unable to utilize lactose as a carbon source for their growth. The HPLC analysis results also supported this statement: no decrement in the lactose concentration was observed until the end of the experiment (data not shown).

### 3.4. PHA Production with Two-Stage Methodology

#### 3.4.1. First Stage—Acetic Acid Production 

**Figure 10:**
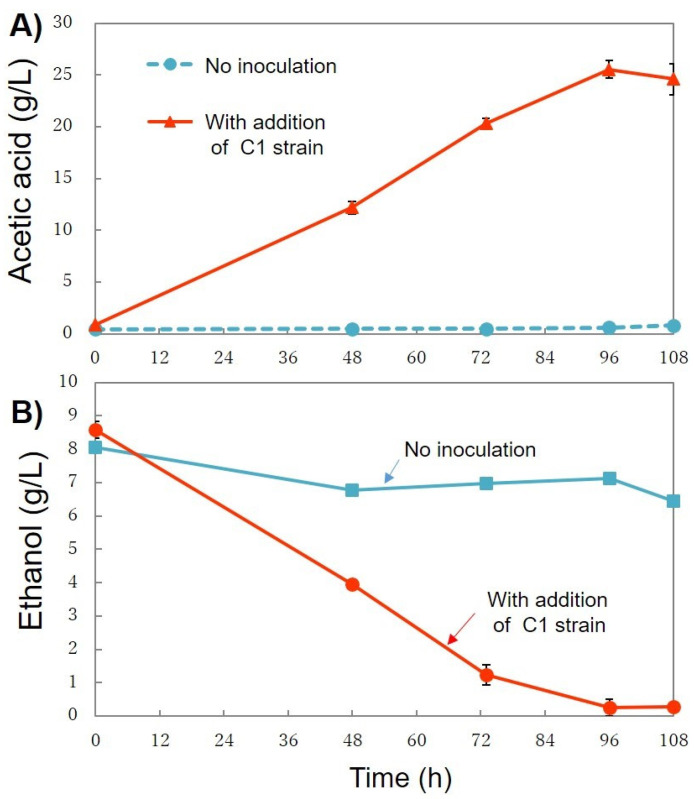
Acetic acid production (**A**) and utilization of ethanol (**B**) by strain C1 using CW (2-fold diluted). CW was used without pre-treatment. All of the conditions were tested as biological triplicates (three Erlenmeyer flasks, *n* = 3) within a single experiment. The data were presented as mean ± standard deviation.

Acetic acid production using the C1 strain with diluted CW was performed. As shown in Figure 10A, the acetic acid concentration (24.4 g/L) gradually increased until 96 h of incubation time. Up to the present day, several reports about acetic acid production with CW have been reported. Lustrato et al. used a yeast (*Kluyveromyces marxianus* Y102) and *Acetobacter aceti* DSM-G3508 co-culture in a bioreactor and produced 4.35 g/L of acetic acid per day under anaerobic conditions [33]. Veeravalli and Mathews produced 24 g/L of acetate under anaerobic conditions using *Lactobacillus buchneri* and CW [34]. *Enterobacter aerogenes* MTCC 2822 immobilized on a column reactor produced 2.1 g/L of acetic acid under anaerobic conditions [35]. Pandey et al. reported that 7 g/L of acetic acid was produced under anaerobic condition by *Lactobacillus acidophilus* in a bioreactor using CW [36]. Ricciardi et al. reported that 1.23 g/L of acetic acid was produced under aerobic conditions by *Lactobacillus casei* in a bioreactor using CW [37]. Gullo et al. reported that acetic acid bacteria can produce acetic acid in fermenting liquids, in which, the ethanol content ranges from 2–3% to 15–18%, depending on the fermentation system and the microbial strains used [20]. As shown in Figure 10B, the ethanol concentration decreased as the acetic acid concentration increased. After 108 h, cultivation was terminated, as the ethanol concentration was 0.27 ± 0.26 g/L, and, in addition, no increment in the acetic acid concentration was observed after 96 h. 

The acetic acid concentration at the initial and final stages was calculated, and a decrement was observed under all of the conditions because of its utilization by bacteria (Table 2). The highest acetic acid consumption of 49.1% was observed at a 10-fold dilution, followed by 31.6%, 14%, and 9% at 20-fold, 4-fold, and 2-fold dilutions, respectively. No decrement was observed with the control (without bacteria), indicating that the CYR1 strain utilizes the acetic acid as a carbon source for its growth.

#### 3.4.2. Second Stage—PHA Production

For PHA production, acetic acid produced from acetic-acid-fermented CW at four dilutions (20-fold, 10-fold, 4-fold, 2-fold) was used. Experiments were also conducted with undiluted CW. The initial acetic acid concentration is 3.8 g/L (20-fold), 5.7 g/L (10-fold), 12.3 g/L (4-fold), and 24.4 g/L (2-fold), and the initial TN concentration is 108 mg/L (20-fold), 234 mg/L (10-fold), 509 mg/L (4-fold), and 1077 mg/L (2-fold). Table 3 shows the results of PHA production at each dilution ratio. The highest PHA production of 240.6 mg/L was obtained with CW at the 10-fold dilution (5.7 g/L, acetic acid). 

**Figure 11:**
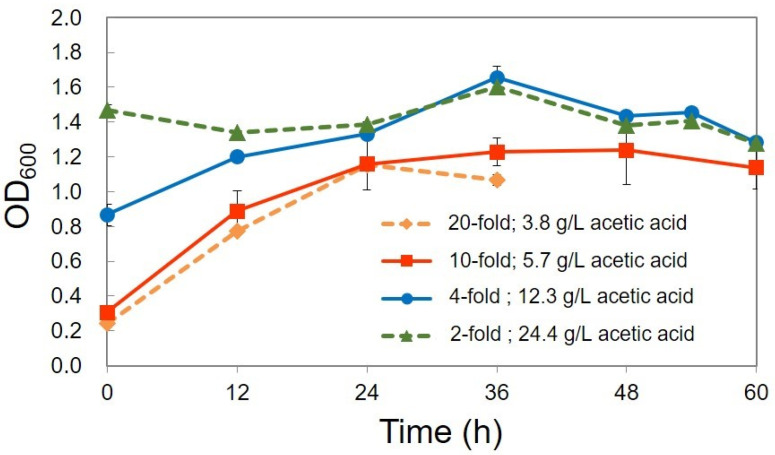
Growth curve of strain CYR1 using acetic-acid-fermented CW without pre-treatment at different dilution rates. All of the conditions were tested as biological triplicates (three Erlenmeyer flasks, *n* = 3) within a single experiment. The data were presented as mean ± standard deviation.

Figure 11 shows the growth of CYR1 with acetic-acid-fermented CW at different dilution rates. The strain CYR1 with undiluted acetic-acid-fermented CW did not show any growth. The strain CYR1 grown with acetic-acid-fermented CW at 4-fold dilution (12.3 g/L, acetic acid) showed growth until 36 h, reached the maximum OD value of 1.66, and then decreased. The strain CYR1 grown with acetic-acid-fermented CW at 10-fold dilution (5.7 g/L, acetic acid) showed growth until 48 h, reached the maximum OD value of 1.33, and then decreased; therefore, the culture was collected at 60 h for PHA extraction. With the 20-fold dilution (3.8 g/L, acetic acid) of CW, the maximum OD value (1.24) was reached at 24 h, which then decreased, so the PHA was extracted at 36 h. The difference in the increase in OD_600_ (final OD–initial OD) was 0.93, 0.91, and 0.79 at 10-fold (5.7 g/L, acetic acid), 20-fold (3.8 g/L, acetic acid), and 4-fold dilutions (12.3 g/L, acetic acid), respectively.

PHA production was carried out with sterilized raw CW and acetic-acid-fermented CW without pre-treatment (Table 4). Koller et al. [38] reported that the sterilization of CW is crucial for PHA production using pure cultures. CYR1 incubated with sterilized CW without acidification at 30-fold dilution produced 46.6 mg of PHA (Table 4). Commercially available acetic acid at various concentrations (5–30 g/L) was also used to produce PHA, where the results showed that the bacteria produced similar amounts of PHA with commercial acetic acid and acetic acid produced from CW (Table 4). Bacteria incubated at a 10 g/L concentration showed a higher PHA production (287 mg/L) than other concentrations. Interestingly, bacteria incubated with acetic acid produced from CW showed a higher PHA production (240.6 mg/L) at 5.7 g/L of the initial acetic acid concentration.

##### Effect of Nitrogen Concentration on PHA Production

An effective PHA production was observed under the low nitrogen concentrations (100 mg/L and 500 mg/L, data not shown); if the nitrogen concentration increased beyond 500 mg/L, the PHA production decreased. This phenomenon was also observed in our previous experiments [39,40]. Our previous studies demonstrated that the gene *PhaC* expression in *Cupriavidus* sp. CY-1 under nitrogen stress was up-and down-regulated with varying nitrogen concentrations [41]. Our previous studies also demonstrated that the strain CYR1 contains four genes, *phaA*, *phaB*, *phaC*, and *phaJ*, which encode the enzymes β-ketothiolase, acetoacetyl-CoA reductase, PHA synthase, and enoyl-CoA hydratase, respectively, which are involved in PHA production [11]. Nitrogen limitation enhances the PHA production in some bacteria because cells cannot grow under the low nitrogen concentration, and try to retain by accumulating PHA, a storage substance of carbon source, for their survival [42,43]. This is why a high PHA production was observed at a 200-500 mg/L nitrogen concentration in our experiments. Apart from the nitrogen concentration, the phosphorus concentration and osmotic pressure of the culture solution might also affect the growth of cells and PHA production [44,45,46,47,48,49,50].

##### PHA Production with Pretreated CW

A COD and TN removal of 24.6% and 28.2% was observed after the pretreatment of raw CW. PHA production experiments were performed using the two-stage process using pretreated CW. As a result, the PHA production was enhanced by 3.26 times (411 mg/L) after pretreatment with the four-fold dilution of fermented CW (Table 5).

All of the conditions were tested as biological duplicates (two Erlenmeyer flasks, *n* = 2) within a single experiment. Data are the means values of two samples.

The PHA production (%CDW) was increased by approximately two times after the pretreatment of CW at all of the dilution rates. It is easy to work with whey supernatants because they are sterile, homogeneous, and straightforward solutions, and produce more PHA than whey powder and whey permeates [6].

The disposal of CW is a major problem that all of the dairy industries are facing, and the large amounts of CW are disposed of as a waste material [51,52,53]. Hence, our studies focused on the utilization of whey as an inexpensive carbon source for acetic acid production and the usage of this acetic acid as a potential carbon source for PHA production. As mentioned above, a higher amount of PHA was obtained from acetic-acid-fermented CW rather than whey itself as a raw carbon source. On the other hand, several issues need to be resolved for the commercialization of PHA. The first issue is higher costs, which are associated with the multi-stage production process and the maintenance of the reactors; however, this can be overcome by improving the production efficiency. The second issue is the improvement of PHA yields from CW, which contains lactose. In order to solve this problem, the hydrolysis of lactose into monosaccharides should be considered. If lactose can be efficiently used as a carbon source for bacterial growth, PHA production will be improved. However, our experimental results in this study suggested that the CYR1 strain cannot utilize lactose as a carbon source for its growth. 

The use of enzymatic hydrolysis significantly increases the PHA production cost, and so should be avoided from an industrial point of view. These problems can be avoided by deploying genetic engineering techniques. Therefore, *E. coli* cells that are capable of consuming lactose were modified to express PHA biosynthesis genes from high PHA-producing microorganisms [54,55,56]. Alternatively, high PHA-producing strains were engineered to express lactose degradation genes [57]. Recombinant *E. coli* has commonly been employed for PHA production due to its convenience for genetic manipulation, fast growth, high cell density cultivation, and its ability to utilize inexpensive carbon sources [58]. Therefore, the development of recombinant *E. coli* will be one of the applicable methods to improve PHA production. The third issue is to improve the efficiency of acetic acid utilization. As shown in Table 2, the concentration of acetic acid consumed by the CYR1 strain is 1.0 to 3.0 g/L, regardless of its initial concentration. A large amount of acetic acid remained unused by bacteria; at the moment, we do not know the exact reason. If bacteria can utilize more acetic acid for their growth, the PHA production will improve. Recently, Chen et al. reported the metabolic engineering of *E. coli* for the synthesis of PHA using acetate as the primary carbon source [59]. The overexpression of the phosphotransacetylase/acetate kinase pathway was shown to be an effective strategy for improving acetate assimilation and biopolymer production. The recombinant strain overexpressing the phosphotransacetylase/acetate kinase and PHB synthesis operon produced 1.27 g/L PHB when grown on a minimal medium supplemented with 10 g/L yeast extract and 5 g/L acetate in shake flask cultures. Thus, the metabolic engineering of *E. coli* will be one of the applicable methods to improve PHA production. The fourth issue is that the production cost of plastics from petrochemical products is still more competitive, and chemical synthesis is preferred by companies [60]. To overcome these problems, research must focus on the production of PHA from CW. 

### 3.5. Identification of PHB Produced from the Two-Stage Process 

**Figure 12:**
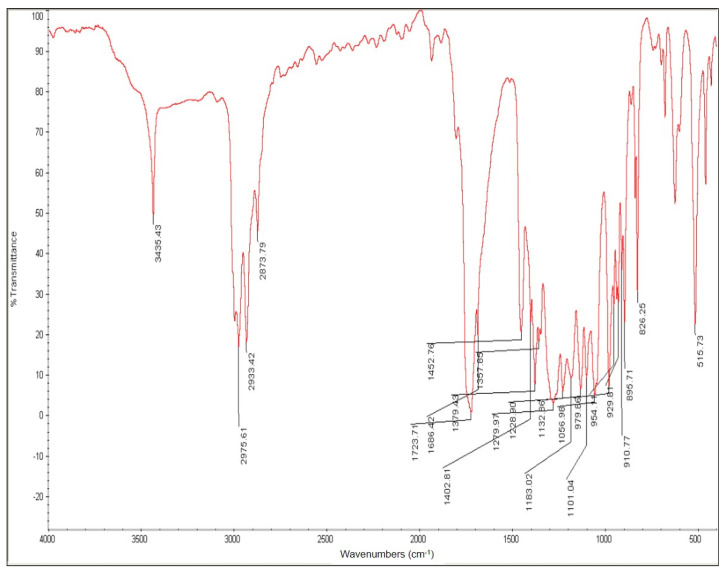
FT-IR spectra of PHB extracted from fermented CW containing 5.7 g/L of acetic acid. The fermented CW is obtained without pre-treatment of CW.

The band appearing in the FT-IR spectrum at 1453 cm^−1^ matches the asymmetrical deformation of the C–H bond within CH_2_ groups, and CH_3_ groups at 1379 cm^−1^ (Figure 12). The band at 1724 cm^−1^ corresponds to the stretching of the C=O bond, whereas a series of intense bands located at 1000–1300 cm^−1^ corresponds to the stretching of the C–O bond of the ester group. All bands in the sample are identical to those of standard PHB. The methylene C–H vibration near 2933 cm^−1^ is also observed. The presence of absorption bands at 1724 cm^−1^ and 1280 cm^−1^ in the extracted PHB sample are characteristic of C=O and C–O, respectively. 

**Figure 13:**
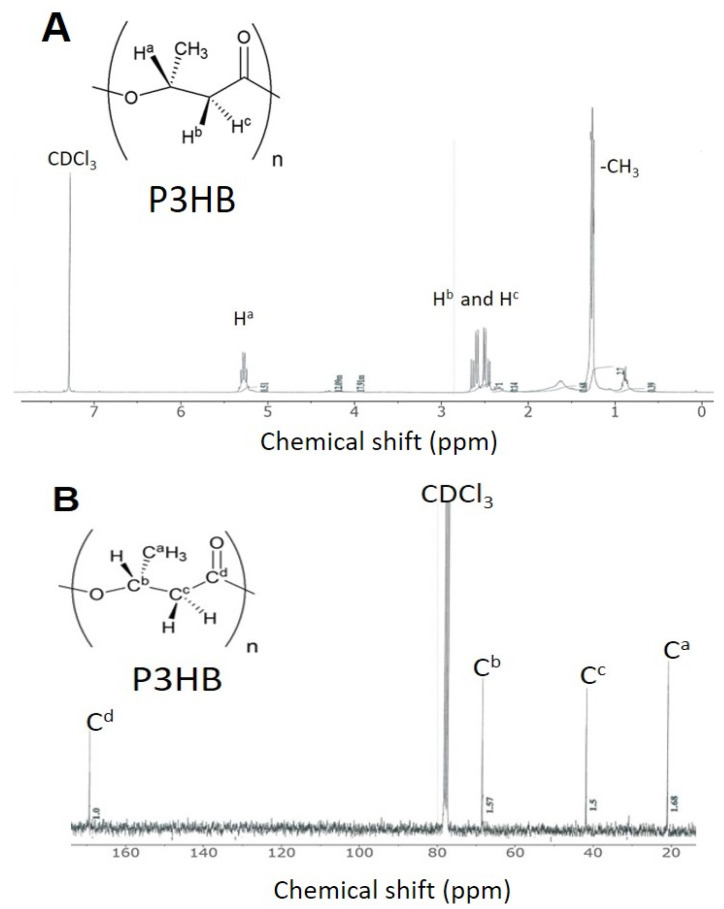
(**A**) ^1^H and (**B**) ^13^C NMR spectra of PHB extracted from fermented CW containing 5.7 g/L of acetic acid. The fermented CW is obtained without pre-treatment of CW.

The ^1^H NMR and ^13^C NMR spectra of the CDCl_3_-soluble part of PHB, extracted from CYR-1 grown with fermented CW containing acetic acid (5.7 g/L), were measured at 20 °C. Based on their peak positions, each peak was assigned to the protons on methine (5.25 ppm), methylene (2.64–2.43 ppm), and methyl (1.25 ppm) groups in the ^1^H NMR spectrum (Figure 13A). The peaks in the ^13^C NMR spectrum (Figure 13B) were assigned to the carbon of the carbonyl (169.36 ppm), methine (67.82), methylene (41.88 ppm), and methyl (20.92 ppm) groups. The chemical shifts of these peaks were nearly identical to those of standard PHB previously reported by Reddy et al. [10] and Chang et al. [61].

**Figure 14:**
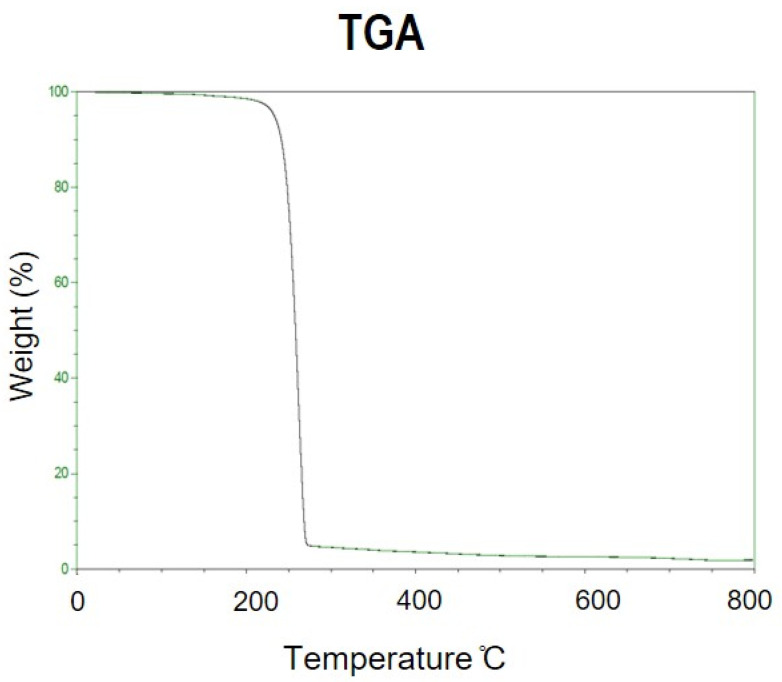
TGA thermograms of PHB extracted from fermented CW containing 5.7 g/L of acetic acid. The fermented CW is obtained without pre-treatment of CW.

In the TGA thermogram, the initial weight loss temperature of the PHB produced by strain CYR1 is at ~170 °C, and its Td is at 200 °C, with its decomposition incomplete until 800 °C (Figure 14). The characteristics of the PHB produced from the two-stage process were comparable to standard PHB.

PHB is synthesized and stored by a wide variety of bacteria, such as *Bacillus* sp. and *Pseudomonas* sp., through the fermentation of various substrates [62]. Among the pure cultures, the bacteria Bacillus strains have been widely studied because of their potential in producing significant amounts of PHB from organic carbon substrates, such as glycerol, dairy wastes, agro-industrial wastes, food industry waste, fatty acids, toxic chemical compounds, and sugars [11,63,64,65,66,67,68]. 

As shown in Table 6, the amount of PHB accumulation from Bacillus strains can wildly vary from 17% to 89% of its dry cell weight, depending on the type and concentration of the substrate [5,64,65,66,67]. *Bacillus aryabhattai* T34-N4 hydrolyzed the cassava pulp and oil palm trunk starch and accumulated up to 17% PHB of the dry cell weight [69]. Moreover, *Bacillus cereus* EGU3 was reported to yield up to 66.6% (0.5 g/L) of PHB content when using contaminated food as a carbon source [70]. Hassan et al. reported that *Bacillus subtilis* yielded up to 62.6% (0.81 g/L) of PHB using rice bran [71]. *Bacillus* sp. N-2 was reported to yield up to 20% (0.17 g/L) of PHB content when using glucose as a carbon source [64]. *Bacillus megaterium* VB89 produced 36.17% (0.67 g/L) of PHB using slurry from the Nisargruna biogas plant [63]. *Bacillus drentensis* BP17 hydrolyzed pineapple peel and accumulated 5.55 g/L of PHB dry cell weight [72]. *Bacillus megaterium* strain A1 isolated from hydrocarbon-contaminated soil produced 48.7% of PHB using molasses [73]. *Bacillus megaterium* S29 and *Bacillus* sp. IPCB-403 accumulated over 70% PHB content per dry cell weight in optimal conditions and using glucose and activated sludges, respectively [74,75]. On the other hand, the strain *Bacillus* sp. CYR1 used in this study produced the highest PHB production with phenol (51%; 0.51 g/L), naphthalene (42%; 0.42 g/L), 4-chlorophenol (32%; 0.32 g/L), and 4-nonylphenol (29%; 0.29 g/L) [67]. *Bacillus firmus* NII 0830 yielded up to 89% (1.9 g/L) of PHB using rice straw [76,77]. *Bacillus cereus* CFR06 produced up to 48% (0.48 g/L) of PHB using starch-based materials [78]. *Bacillus megaterium* produced 26% (0.47 g/L) of PHB using lactose [5]. *Bacillus cereus suaeda* B-001 produced 55.4% (0.47 g/L) of PHB using oil palm empty fruit bunch hydrolysates [79]. *Bacillus flexus* Azu-A2 produced 20.96% (0.95 g/L) of PHB using CW [80]. *Bacillus megaterium* B-10 produced 32.56% (1.496 g/L) of PHB using dilute acid pretreatment liquor of lignocellulose containing abundant waste sugar resource from rice straw [81]. *Bacillus megaterium* strain CAM12 produced 51% (8.31 g/L) of PHB using finger millet straw hydrolysates [82]. *Bacillus tequilensis* PSR-2 produced 49.12% (2.8 g/L) of PHB using glucose (1%) [83]. In addition, the strain PSR-2 could produce a maximum of 12.4 g/L of PHB using an alkali-pretreated spent mushroom substrate of sugarcane bagasse [83]. *B. cereus* 2156 produced 2.2 g/L of PHB using sugarcane molasses [84]. *Bacillus* sp. produced 56% (5.0 g/L) of PHB using sugarcane bagasse hydrolysates [85]. *Bacillus flexus* ME-77 produced 15% (4.5 g/L) of PHB using sugarcane molasses [86]. *B. thuringiensis* SBC4 produced 43.95% (0.4 g/L) of PHB using glucose and corn cob [87]. Recently, Werlang et al. reported that *Bacillus pumilus* could produce PHB using the hydrolysate of the Arthrospira platensis biomass as carbon sources, in addition to glucose [88]. The average production during the experiments was 1.2 mg of PHB from 0.4 g of bacterial biomass [88]. In this study, *Bacillus* sp. CYR1 yielded 33.5% (0.41 g/L) of PHB through the two-stage process. Therefore, it can be said the productivity of PHB is similar to *Bacillus cereus* CFR06, *Bacillus cereus suaeda* B-001, *Bacillus megaterium,* and *B. thuringiensis* SBC4 [5,78,79,87]. According to Sirohi et al. [68], which was recently reviewed, PHB synthesis can be done from several substrates in aerobic/anaerobic conditions and at different temperatures, pH, and states of fermentation, using starch, cellulose, sucrose, molasses, whey, glycerol, and others, which can be present in wastes. In future, we will conduct a study to determine the optimal condition (agitation speed, pH, and temperature) for the production of PHB through the development of the second stage of the process using strain CYR1. In addition to the development of optimal culture conditions, the utilization ability of lactose and acetic acid through molecular engineering methods can be improved; the two-stage process will be adoptable for the industrial process for the production of PHB. 

## 4. Conclusions

A two-stage process was applied to produce PHA from CW containing a low level of lactose using *Acetobacter pasteurianus* C1 and *Bacillus* sp. CYR1. Acetic acid fermentation was effectively conducted using CW by a newly isolated C1 strain. The dilution of acetic-acid-fermented liquid is essential to lower the nitrogen concentration and to improve the PHA production. In addition, the pretreatment of raw CW also enhanced the PHA production. The PHA produced from the two-stage process using CW was PHB. Results discovered that, instead of the hydrolysis of lactose (which enhances the production cost), a two-stage treatment would be applicable for PHA production from CW, which contains a low level of lactose.

## Figures and Tables

**Table 2:** Acetic acid consumption by CYR1 strain using acetic-acid-fermented CW. CW was used without pre-treatment at different dilutions.

Culture Condition	20-Fold	10-Fold	4-Fold	2-Fold
Initial concentration of acetic acid (g/L)	3.8	5.7	12.3	24.4
Final concentration of acetic acid (g/L)	2.6	2.9	10.6	22.2
The utilized acetic acid (g/L)	1.2	2.8	1.7	2.2
The utilized acetic acid (%)	31.6	49.1	13.8	9.0

All of the conditions were tested as biological duplicates (two Erlenmeyer flasks, *n* = 2) within a single experiment. Data are the mean values of two Erlenmeyer samples.

**Table 3:** PHA production after the two-stage process using acetic-acid-fermented CW without pre-treatment.

Culture Condition	20-Fold (Acetic Acid; 3.8 g/L)	10-Fold (Acetic Acid; 5.7 g/L)	4-Fold (Acetic Acid; 12.3 g/L)	2-Fold (Acetic Acid; 24.4 g/L)
CDW (mg)	151.6	319.4	638.2	831.7
PHA production (mg/L)	43.6	240.6	126.2	77.2
PHA production (%CDW)	28.8	75.4	19.8	9.2
Yield of PHA from acetic acid (%)	3.6	8.6	7.4	3.5
PHA volumetric productivity (mg PHA/ L/hour)	1.21	4	2.1	1.28

CDW: cell dry weight; All of the conditions were tested as biological duplicates (two Erlenmeyer flasks, *n* = 2) within a single experiment. Data are the means values of two samples.

**Table 4:** PHA production under each condition using acetic-acid-fermented CW without pre-treatment, raw CW, and pure acetic acid.

Media (Substrates)		Initial Acetic Acid Concentration (g/L)	Extracted PHA (mg/L)
Acetic acid fermented from CW	2-fold	24.4	77.2
	4-fold	12.3	126.1
	10-fold	5.7	240.6
	20-fold	3.8	43.6
One step production of PHA using raw CW	30-fold	not detected	46.6
One step production of PHA using mineral medium containing pure acetic acid	30 g/L	30.0	8.6
	20 g/L	20.0	231.0
	10 g/L	10.0	287.0
	5 g/L	5.0	146.6

All media were sterilized. All of the conditions were tested as biological duplicates (two Erlenmeyer flasks, *n* = 2) within a single experiment. Data are the means values of two samples.

**Table 5:** PHA production of the two-stage process using pre-treated CW at different dilutions.

Culture Condition	20-Fold (Acetic Acid; 2.3 g/L)	10-Fold (Acetic Acid; 4.5 g/L)	4-Fold (Acetic Acid; 11.3 g/L)
CDW (mg)	155.2	261.4	1227.2
PHA production (mg/L)	58.0	96.6	411.2
PHA production (%CDW)	37.4	37.0	33.5

**Table 6:** Overview of PHB production of genus Bacillus using various carbon sources.

Strain	Carbon Source	PHAs Monomer	PHB Conc. (g/L)	PHB Content. (wt%)	References
*Bacillus megaterium*	lactose	PHB	0.47	26.0	[5]
*Bacillus megaterium* VB89	slurry from Nisargruna biogas plant	PHB	0.67	36.17	[63]
*Bacillus* sp. N-2	glucose	PHB	0.17	20.0	[64]
*Bacillus* sp. CYR1	phenol	PHB	0.51	51.0	[67]
*Bacillus* sp. CYR1	naphthalene	PHB	0.42	42.0	[67]
*Bacillus* sp. CYR1	4-chlorophenol	PHB	0.32	32.0	[67]
*Bacillus* sp. CYR1	4-nonylphenol	PHB	0.29	29.0	[67]
*Bacillus aryabhattai* T34-N4	oil palm trunk starch	PHB	0.33	17.0	[69]
*Bacillus cereus* EGU3	contaminated food	PHB	0.5	66.6	[70]
*Bacillus subtilis*	rice bran	PHB	0.81	62.6	[71]
*Bacillus drentensis* BP17	pineapple peel	PHB	5.55	n.a	[72]
*Bacillus megaterium* strain A1	molasses	PHB	14.6	48.7	[73]
*Bacillus megaterium* S29	glucose	PHB	n.a	≧70	[74]
*Bacillus* sp. IPCB-403	activated sludges	PHB	n.a	≧70	[75]
*Bacillus firmus* NII 0830	rice straw	PHB	1.9	89.0	[76,77]
*Bacillus cereus* CFR06	starch-based materials	PHB	0.48	48.0	[78]
*Bacillus cereus suaeda* B-001	oil palm empty fruit bunch hydrolysates	PHB	0.47	55.4	[79]
*Bacillus flexus* Azu-A2	cheese whey	PHB	0.95	20.96	[80]
*Bacillus megaterium* B-10	dilute acid pretreatment liquor of lignocellulose from rice straw	PHB	1.496	32.56	[81]
*Bacillus megaterium* strain CAM12	finger millet straw hydrolysates	PHB	8.31	51.0	[82]
*Bacillus tequilensis* PSR-2	glucose	PHB	2.8	49.12	[83]
*Bacillus tequilensis* PSR-2	an alkali-pretreated spent mushroom substrate of sugarcane bagasse	PHB	12.4	n.a	[83]
*B. cereus* 2156	sugarcane molasses	PHB	2.2	n.a	[84]
*Bacillus* sp.	sugarcane bagasse hydrolysates	PHB	5.0	56.0	[85]
*Bacillus flexus* ME-77	sugarcane molasses	PHB	4.5	15.0	[86]
*Bacillus thuringiensis* SBC4	glucose and corn cob	PHB	0.4	43.95	[87]
*Bacillus pumilus*	hydrolysate of the Arthrospira platensis biomass	PHB	n.a	0.3	[88]
*Bacillus* sp. CYR1	cheese whey	PHB	0.41	33.5	This study

n.a.: not avaiable.

## Data Availability

Not applicable.
